# Effect of sagittal screw angle and distance of screw apex to superior endplate on adjacent segment disease after posterolateral lumbar fusion: a retrospective study

**DOI:** 10.1186/s13018-022-03383-z

**Published:** 2022-11-16

**Authors:** Qiang Wang, Zhiqiang Gao, Kai Guo, Feng Wang, Desheng Wu

**Affiliations:** Department of Spine Surgery, Shanghai East Hospital, Tongji University School of Medicine, 150 Jimo Rd., Shanghai, 200120 China

**Keywords:** Adjacent segment disease, Sagittal screw angle, Distance, Visual analogue score, Oswestry disability index

## Abstract

**Background:**

Numerous complications of lumbar fusion surgery have been reported, with adjacent segment disease (ASD) being one of the most important. Few studies describe the effect of sagittal, horizontal screw angles and distance of pedicle screw apex to superior endplate on the incidence of ASD in lumbar spine. The purpose of this retrospective study is to evaluate the hypothesis that unsatisfactory pedicle screw insertion positions would increase the likelihood of ASD.

**Methods:**

Outpatients with lumbar spinal stenosis underwent posterolateral lumbar fusion at L4-S1 with a least 2-year follow-up were studied. ASD at L3–L4 was defined as a condition in which intervertebral disk narrowing, posterior vertebral opening, and vertebral slippage progress at the last follow-up in comparison with the postoperative. Independent *t* test was performed to compare data between two groups; Spearman analysis was performed to analyze the relationship between two continuous variables. Multivariate binary logistic models were performed to identify the independent risk factors of ASD. The receiver operating characteristic (ROC) curve was performed to measure model discrimination and Hosmer–Lemeshow (H–L) test was used to measure calibration. ROC curve evaluated the discrimination ability of sagittal screw angle and distance in predicting incidence of ASD.

**Results:**

Patients in ASD group exhibit higher incidence of osteoporosis, higher Visual analogue scale (VAS), Oswestry disability index (ODI), bigger sagittal screw angle, shorter distance of pedicle screw apex to superior endplate than those in non-ASD group (*p* < 0.05). VAS, ODI at the last follow-up were positively correlated with Pfirrmann grade of L3–4 disk and sagittal screw angle, while negatively correlated with distance of screw apex to superior endplate (*p* < 0.05). Multivariate binary logistic model indicated that follow-up time (odds ratio [OR] 1.637, 95% confidence interval [CI] 1.186–2.260), distance of screw apex to superior endplate (OR 0.150, 95% CI 0.067–0.336), sagittal screw angle (OR 2.404, 95% CI 1.608–3.594) were statistically significant. The models showed great discrimination and calibration. The area under the curve of ASD identified by sagittal angle and distance was 0.895 and the cut-off values were 5.500° and 6.250 mm, respectively.

**Conclusion:**

Sagittal screw angle and distance of screw apex to superior endplate were significantly associated with the risk of ASD.

## Background

Various complications and problems related to PLF have been reported. Among these complications, the incidence of radiographical ASD has been reported to be higher than 8%, attributing to the fact that constrained mobility and loads of the instrumented segments is compensated by the adjacent segment [1, 2]. Most degenerative change occurred in the proximal segment to the fusion levels and patients with ASD may require revision surgery [3, 4]. In addition to known risk factors of ASD including old age, female sex, high body mass index (BMI), osteoporosis, fusion length, violation of adjacent facet during surgery, pre-existing adjacent intervertebral disk degeneration and sagittal imbalance [5–8]. In fact, even when pedicle screws do not violate superior facet joints, pedicle screw fixation imposes excessive stress on vertebral body and adjacent intervertebral disk [9]. In the outpatient follow-up, we found that patients with persistent low back pain after PLF were accompanied by bigger sagittal cranial oriented screw angle and shorter distance between the pedicle screw apex and superior endplate. The relationship between sagittal screw angle and stress on endplate of adjacent segment after anterior cervical corpectomy and fusion has been reported [10]. However, few studies are available that describe the effect of varying sagittal, horizontal screw angles and distance of pedicle screw apex to superior endplate on the incidence of ASD in lumbar spine. Accordingly, the purpose of this retrospective study is to evaluate the hypothesis that unsatisfactory pedicle screw insertion positions would increase the likelihood of superior ASD.

## Materials and methods

### Patients’ population

This study was conducted based on the principle of voluntary participation and was in accordance with the protocols proposed by the Ethic committees of Shanghai East Hospital. All the patients signed corresponding informed consents and verbally informed prior to the study and understood the purpose for which their data will be used. Four-hundred and forty-three patients who underwent PLF at L4-S1 combined with or without bone cement screw at L5 by one surgeon in our hospital and underwent outpatient follow-up between September 2015 and June 2022. Finally, 189 patients with at least 2 years of postsurgical follow-up were included in our study. The inclusion criteria were the presence of preoperative lumbar spinal stenosis at L4-S1 levels, chronic and persistent radiculopathy despite conservative treatment, progressive neurological deficits, persistent and unremitting low back pain for more than 6 months, loss of quality of life because of neurological claudication. The exclusion criteria were as follows: spondylolisthesis, concomitant scoliosis of more than 15°, violation of adjacent facet during surgery, patients with prior spinal surgery, postoperative infection and patients who underwent implant removal during the follow-up period.

### Surgical process

In all patients, PLF at L4-S1 were performed. After a midline surgical exposure, the soft tissues were dissected to the lateral tips of the transverse processes. Exposure for the superior facet joint required detachment of muscle from facet capsule, but care was taken to preserve the capsule itself. During surgery, pedicle screw and rod instrumentation were performed without violating the facet joint.

### Demographics and radiological evaluation

**Fig. 1:**
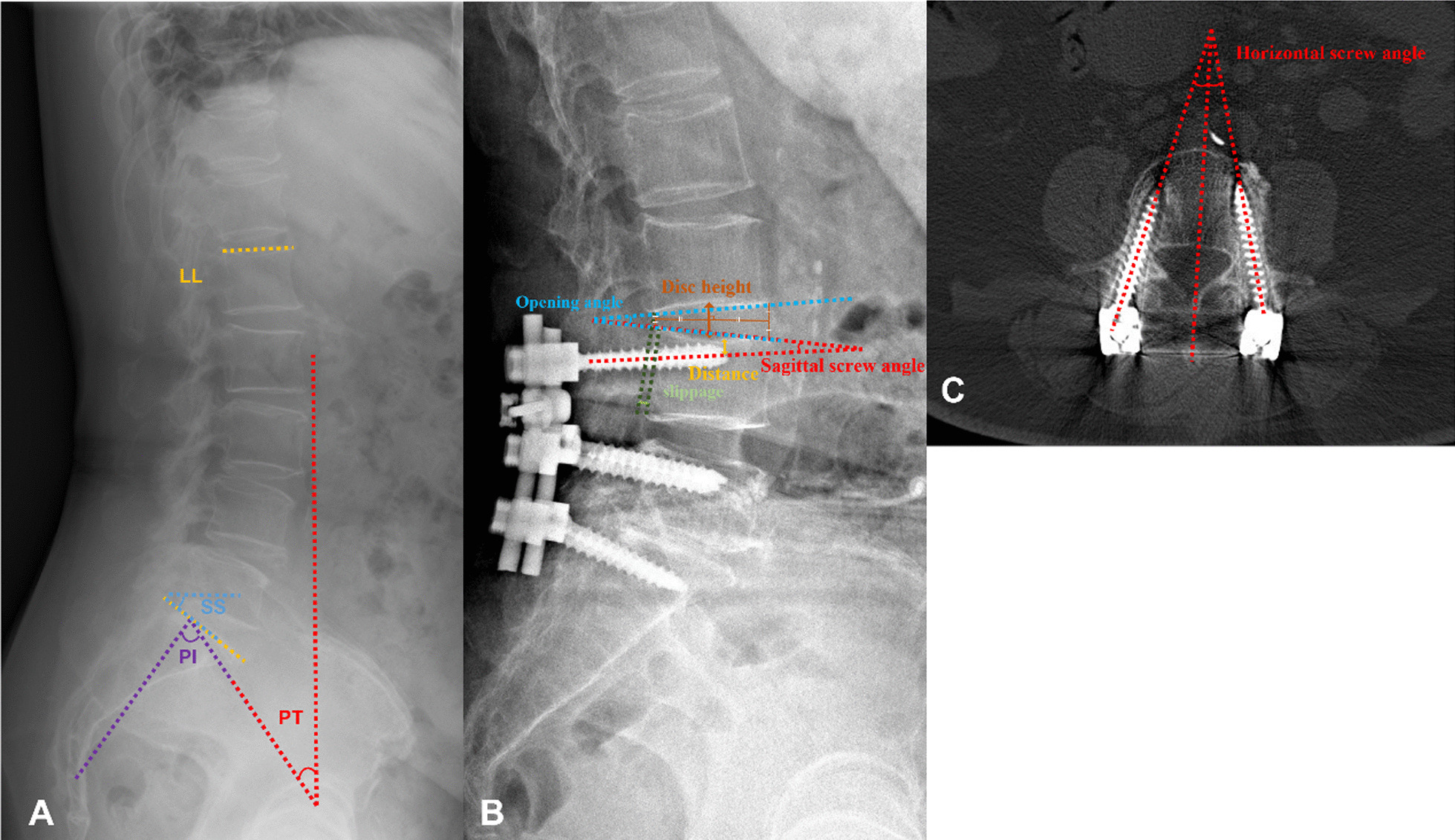
Parameters of radiological evaluation of spinopelvic parameters, ASD diagnosis and pedicle screw fixation. **A** Preoperative spinopelvic parameters of patients evaluated by lateral radiograph. **B** Proximal adjacent segment disk height, opening angle at L3–4, L3 vertebral slippage, distance of pedicle screw apex to superior endplate and sagittal screw angle between pedicle screw and superior endplate of L4 by lateral radiograph (not the same patient in **A**). **C** Horizontal screw angle, the mean angle of two pedicle screws at L4 and middle line by horizontal plane

Preoperative demographic data including gender, age, body mass index (BMI), osteoporosis, years of follow-up were collected. Clinical outcomes including Oswestry Disability Index (ODI), low back visual analogue scale (VAS) at the last follow-up were recorded. Radiological evaluation including spinopelvic parameters, pedicle screw related, and ASD diagnosis-related parameters were measured by lateral radiographs. Spinopelvic parameters including sacral slope (SS), pelvic tilt (PT), lumbar lordosis (LL), pelvic incidence minus lumbar lordosis (PI-LL) were measured preoperatively [11] (Fig. 1A). Pedicle screw-related parameters including postoperative sagittal screw angle (mean angle between two pedicle screws and the superior endplate of L4, expressed it as “+” in screws angled cranially and “−” in screws angled caudally), horizontal screw angle (Fig. 1C, mean angle between two pedicle screws and middle of the line at horizontal plane, measured by 3D reconstruction of computed tomographic CT scans), distance (vertical line connecting the front and rear edges of the upper endplate passes the distance from the screw apex to the superior endplate) (Fig. 1B). ASD diagnosis-related parameters including intervertebral disk height, opening angle at L3–4 and vertebral slippage of L3 were measured by lateral lumbar radiographs. Intervertebral disk height was defined as equidistant on the line drawn to bisect the middle points of the anterior and posterior disk height [12] (Fig. 1B). L3 vertebral slippage is distance between the lines of the posterior upper and posterior lower edges of L4 and the parallel lines passing through the posterior lower edge of L3 (parallel lines passing through the posterior lower edge of L3 vertebral body were + in the anterior distance and − in the posterior distance) (Fig. 1B).

**Fig. 2:**
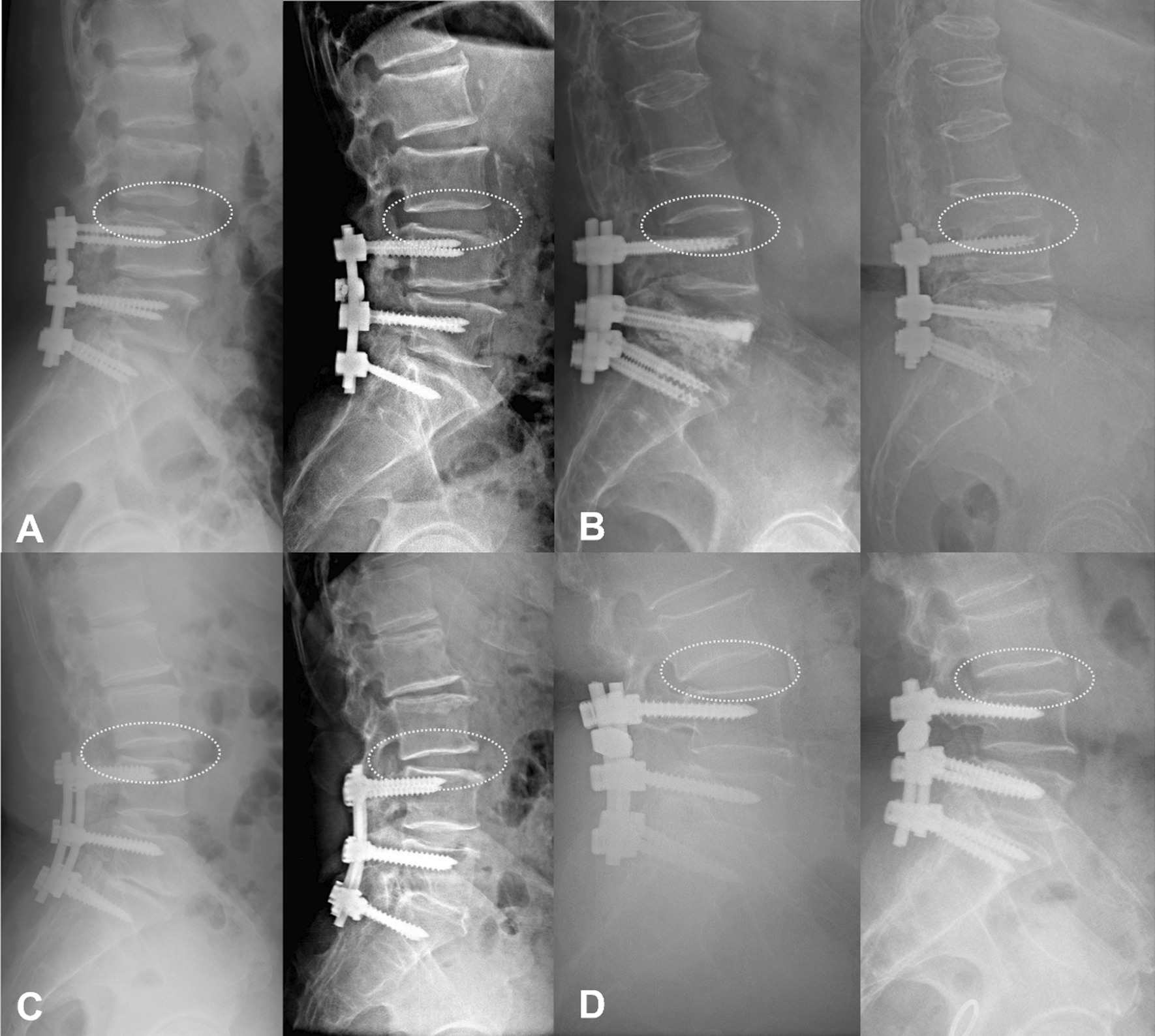
Comparison of ASD diagnosis-related parameters perioperatively and at the last follow-up by lateral radiograph. **A** non-ASD patient without pathological adjacent vertebral and intervertebral disk changes. **B** Narrowing of disk height (> 3 mm) accompanied by wedge compression of the upper vertebral body. **C** posterior slippage (> 3 mm) of L3 vertebral. **D** posterior opening at L3–4 in the flexion position after surgery (> 5°)

Radiographical ASD was diagnosed at the last follow-up when plain radiographs demonstrated one or more of the following lesions at the segment adjacent to the fused segments relative to postoperative: narrowing of disk height (> 3 mm), anterior or posterior slippage (> 3 mm), posterior opening between adjacent vertebral bodies (> 5°) [13] (Fig. 2). Preoperative disk degeneration on magnetic resonance imaging was rated from grades 1 to 5 using the classification system of Pfirrmann [14]. Diagnosis of osteoporosis was referred to Mu et al. [15], dual-energy X-ray absorptiometry (DXA) was used to collect the average L1–4 vertebral bone mineral density (BMD) value, set *T* value >  − 2.5 as negative and *T* value ≤  − 2.5 as osteoporosis. Two orthopedic spine surgeons, who were not involved with the operation and blinded to all clinical information, performed radiological measurement using picture archiving and communication system. The measurements were performed twice for each parameter with an adequate time interval to prevent bias from distorting the results. To minimize the interobserver error, average values were used for statistical analyses.

### Statistical analysis

Statistical analysis was performed using SPSS 17.0 statistical package (SPSS, Inc., Chicago, IL). The descriptive results were expressed as mean and standard deviation (SD) for continuous variables with an approximately normal distribution. The independent t test was performed to compare demographic, radiological, and clinical data between the two groups, Spearman analysis was performed for correlation between clinical evaluation and characteristics of patients. Two multivariable logistic regression models were obtained for continuous/two categorical indicators (sagittal screw angle and distance) with a binary indicator of ASD as the dependent variable and clinical characteristics as the candidate independent variables. Hosmer–Lemeshow goodness of fit test was used to assess the model fit (Hosmer–Lemeshow statistic ≥ 0.05). Sagittal screw angle and distance were tested to discriminate ASD using receiver operating characteristic (ROC) curves. Youden is defined as the sum of specificity + sensitivity − 1. *p* value of less than 0.05 was accepted as significant.

## Results

### Characteristics of the patients

One hundred and eighty-nine patients were included with 53 patients in ASD group and 136 patients in non-ASD group with at least 2-year radiological and clinical follow-up data. The mean age of these patients at the time of surgery was 61 years (range 27–81 years). No major surgery-related complications occurred, including wound infection, additional neurological dysfunction, or hardware failure. The gender, age, BMI, years of follow-up, Pfirrmann degree at L3–4, horizontal screw angle and spinopelvic parameters were comparable between two groups (*p* > 0.05). ASD group had a significantly higher low back VAS (6.7 ± 1.5 vs 5.1 ± 1.1, *p* < 0.001), higher ODI (20.0 ± 3.7 vs 14.4 ± 3.2, *p* < 0.001), bigger sagittal screw angle (5.7 ± 1.2 vs 4.5 ± 1.1, *p* < 0.001), higher incidence of osteoporosis (24.5% vs 7.4%, *p* = 0.001), while ASD group had a significantly shorter distance of pedicle screw apex to superior endplate (6.0 ± 0.6 vs 6.8 ± 0.8, *p* < 0.001) compared with non-ASD group (Table 1).

**Table 1:** Characteristics of patients

	Non-ASD (*n* = 136)	ASD (*n* = 53)	*p*
Gender (female/male)			
Female	62	27	
Male	74	26	0.510
Age (yr)	60.5 ± 8.7	61.3 ± 9.1	0.574
BMI (Kg/m^2^)	25.3 ± 1.5	25.2 ± 1.2	0.730
Follow-up (yr)	3.5 ± 1.5	3.8 ± 1.4	0.108
Osteoporosis			
Without	126	40	
With	10	13	**0.001**
Pfirrmann			
Grade 1	1	0	
Grade 2	25	3	
Grade 3	64	30	
Grade 4	46	20	0.109
Distance (mm)	6.8 ± 0.8	6.0 ± 0.6	< **0.001**
Horizontal screw angle (°)	16.3 ± 2.4	16.4 ± 2.0	0.861
Sagittal screw angle (°)	4.5 ± 1.1	5.7 ± 1.2	< **0.001**
VAS	5.1 ± 1.1	6.7 ± 1.5	< **0.001**
ODI	14.4 ± 3.2	20.0 ± 3.7	< **0.001**
PI-LL (°)	18.3 ± 6.6	18.3 ± 6.6	0.987
SS (°)	31.9 ± 4.5	31.8 ± 2.9	0.862
PT (°)	22.1 ± 4.4	23.0 ± 3.0	0.166
PI (°)	54.0 ± 5.9	54.8 ± 4.8	0.385

BMI, Body mass index; VAS, Visual analogue scale; ODI, Oswestry disability index; Distance, The distance between pedicle screw apex and superior endplate; SS, Sacral slope; PT, Pelvic tilt; PI-LL, Pelvic incidence minus lumbar lordosis

Boldface values indicate *p* < 0.05

Comparison of ASD diagnosis parameters between the ASD and the non-ASD group postoperatively and at the last follow-up are shown in Table 2. The ASD group had a significantly more narrow intervertebral disk height (5.9 ± 1.5 vs 7.3 ± 1.2, *p* < 0.001), bigger posterior opening at L3–L4 vertebral (4.3 ± 0.9 vs 3.1 ± 0.7, *p* < 0.001) and more severe posterior slippage (− 3.0 ± 1.8 vs − 1.8 ± 1.1, *p* < 0.001) of L3 at the last follow-up. While intervertebral disk height, opening angle at L3-L4 vertebral and vertebral slippage of L3 showed no significant differences postoperatively between groups (Table 2).

**Table 2:** Comparison of ASD diagnosis parameters between the two groups postoperatively and at the last follow-up

	Non-ASD	ASD	*p*
Postoperative disk height	8.6 ± 1.1	8.7 ± 1.2	0.872
Follow-up disk height	7.3 ± 1.2	5.9 ± 1.5	< **0.001**
Postoperative slippage	− 2.4 ± 1.2	− 2.1 ± 1.1	0.180
Follow-up slippage	− 1.8 ± 1.1	− 3.0 ± 1.8	< **0.001**
Postoperative posterior opening	2.9 ± 0.7	2.9 ± 0.7	0.896
Follow-up posterior opening	3.1 ± 0.7	4.3 ± 0.9	< **0.001**

*ASD* Adjacent segment disease

Boldface values indicate *p* < 0.05

### Influencing factors of clinical outcome

To reveal the influencing factors of clinical outcome, correlation between clinical outcome and patient characteristics are correlated in Table 3. At the last follow-up, low back VAS was positively correlated with incidence of osteoporosis (*r* = 0.171, *p* = 0.019), Pfirrmann grade (*r* = 0.173, *p* = 0.018) and sagittal screw angle (*r* = 0.294, *p* < 0.001), while negatively correlated with distance (*r* =  − 0.183, *p* = 0.012) and SS (*r* =  − 0.253, *p* < 0.001). Besides, ODI was positively correlated with Pfirrmann grade (*r* = 0.236, *p* = 0.001) and sagittal screw angle (*r* = 0.329, *p* < 0.001), while negatively correlated with distance (*r* =  − 0.213, *p* = 0.003).

**Table 3:** Correlation between clinical outcome and patient characteristics

	VAS	ODI
Gender	*r* = − 0.020	*r* = − 0.056
	*p* = 0.787	*p* = 0.445
Age (yr)	*r* = − 0.031	*r* = 0.013
	*p* = 0.673	*p* = 0.854
BMI (Kg/m^2^)	*r* = 0.025	*r* = 0.055
	*p* = 0.733	*p* = 0.454
Follow-up (yr)	*r* = 0.044	*r* = 0.125
	*p* = 0.544	*p* = 0.087
Osteoporosis	***r* = 0.171**	*r* = 0.061
	***p* = 0.019**	*p* = 0.403
Pfirrmann	***r* = 0.173**	***r* = 0.236**
	***p* = 0.018**	***p* = 0.001**
Distance (mm)	***r* = ** − **0.183**	***r* = ** − **0.213**
	***p* = 0.012**	***p* = 0.003**
Horizontal screw angle (°)	*r* = − 0.023	*r* = 0.044
	*p* = 0.750	*p* = 0.551
Sagittal screw angle (°)	***r* = 0.294**	***r* = 0.329**
	***p*** < **0.001**	***p*** < **0.001**
PI-LL (°)	*r* = − 0.049	*r* = − 0.019
	*p* = 0.502	*p* = 0.795
SS (°)	***r* = ** − **0.253**	*r* = − 0.141
	***p*** < **0.001**	*p* = 0.053
PT (°)	*r* = − 0.027	*r* = 0.067
	*p* = 0.709	*p* = 0.361

ASD, Adjacent segment disease; Distance, The distance between pedicle screw apex and superior endplate; SS, Sacral slope; PT, Pelvic tilt; PI-LL, Pelvic incidence minus lumbar lordosis

Boldface values indicate *p* < 0.05

### Multivariate analysis of regression

**Fig. 3:**
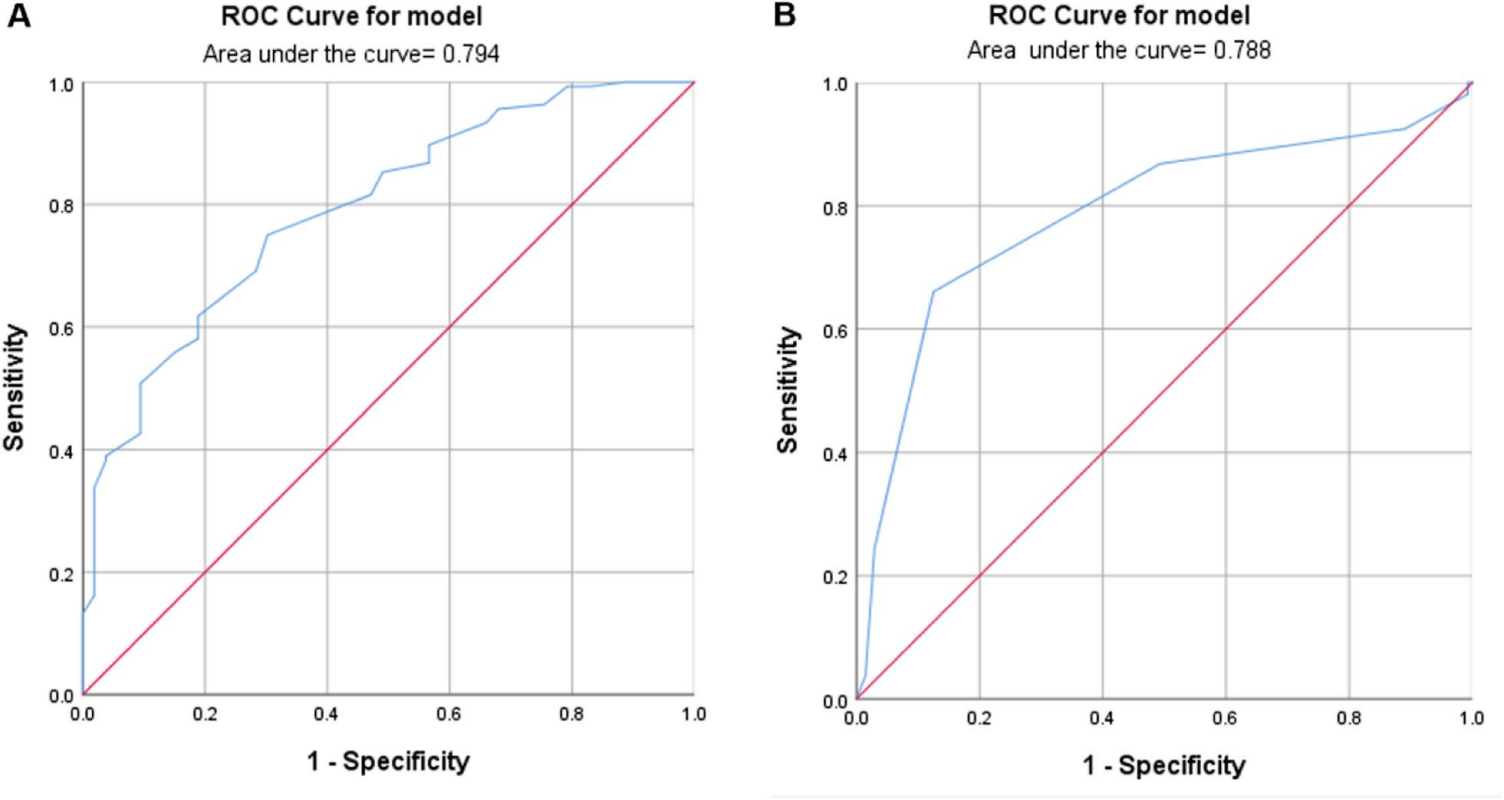
ROC curve of risk factors predicting ASD. A. ROC curve of distance predicting incidence of ASD. The AUC was 0.794. B.ROC curve of sagittal angle predicting incidence of ASD. The AUC was 0.788. *ROC* Receiver operator curve, *AUC* Area under the curve

**Fig. 4:**
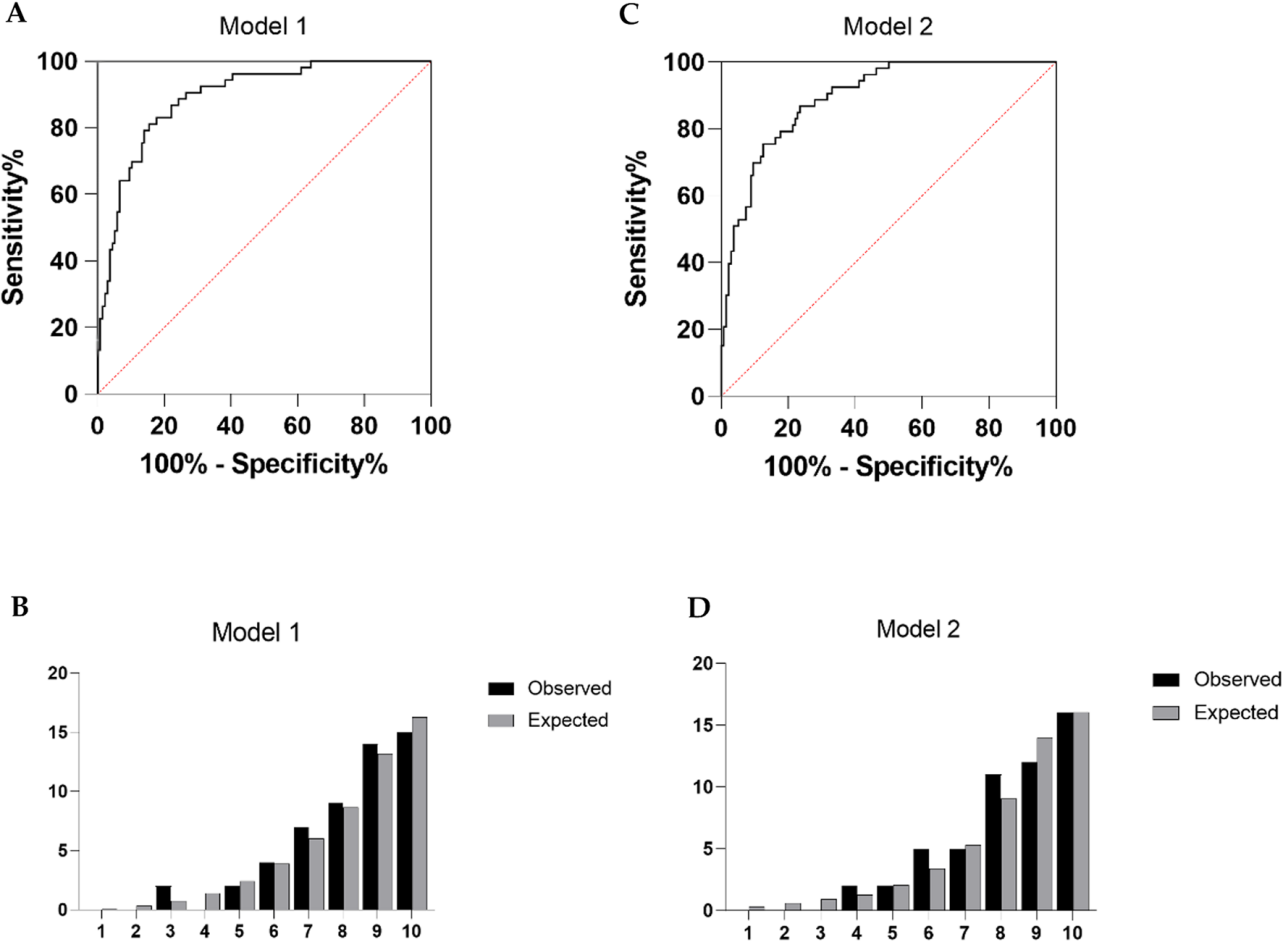
Actual versus predicted ASD by risk deciles for model 1 (**B**) and model 2 (**D**) (see Tables 4, 5 for included variables). ROC curve analysis of prognostic model 1 (**A**) and model 2 (**C**)

Table 4 shows the multivariate logistic regression analysis of risk factors for ASD. The analysis revealed that prolonged follow-up (odds ratio [OR 1.637, 95% confidence interval [CI] 1.186–2.260), shorter distance of pedicle screw apex to superior endplate (OR 0.150, 95% CI 0.067–0.336), bigger sagittal screw angle at L4 (OR 2.404, 95% CI 1.608–3.594) were significantly associated with an increased OR for ASD. ROC curves of distance and sagittal screw angle predicting the incidence of ASD are shown in Fig. 3. The Youden index reaches maximum value when the sagittal angle cut-off is set to 5.500° (sensitivity of 0.66 and specificity of 0.88) and distance cut-off is set to 6.250 mm (sensitivity of 0.75 and specificity of 0.70). Sagittal screw angle and distance were included as continuous variables in model 1. To reveal the actual predictive value of the model. The model 1 was significant, *p* = 0.684 for Hosmer and Lemeshow goodness of fit test. The observed vs predicted risk of ASD in model 1 showed a good fit (Fig. 4B). The area under the ROC curve of model 1 is 0.894 (Fig. 4A). When sagittal angle and distance are divided into categorical variables according to Youden index. Also, the model 2 was significant, *p* = 0.742 for Hosmer and Lemeshow goodness of fit test. The observed vs predicted risk of ASD in model 2 also showed a good fit (Fig. 4D) and the area under the ROC curve of model 2 is 0.895 (Fig. 4C).

**Table 4:** Multivariate analysis Model 1 of possible risk factors for ASD

	*B*	*p*	OR	95% CI for OR	
				Lower	Upper
Gender	0.433	0.349	1.542	0.623	3.822
Age (yr)	0.013	0.617	1.013	0.963	1.065
BMI (Kg/m^2^)	− 0.212	0.213	0.809	0.579	1.130
Follow-up (yr)	0.493	**0.003**	1.637	1.186	2.260
Osteoporosis	1.064	0.090	2.898	0.849	9.896
Pfirrmann	0.421	0.207	1.523	0.792	2.926
Distance	− 1.900	< **0.001**	0.150	0.067	0.336
Sagittal screw angle	0.877	< **0.001**	2.404	1.608	3.594
SS	− 0.006	0.919	0.994	0.887	1.114
PT	0.069	0.211	1.071	0.962	1.194

B, Regression coefficient; OR, Odds ratio; CI, Confidence interval; SS, Sacral slope; PT, Pelvic tilt; PI-LL, Pelvic incidence minus lumbar lordosis

Boldface values indicate *p* < 0.05

**Table 5:** Multivariate analysis Model 2 of possible risk factors for ASD

	*B*	*p*	OR	95% CI for OR	
				Lower	Upper
Gender	0.667	0.167	1.948	0.756	5.017
Age (yr)	0.020	0.413	1.021	0.972	1.072
BMI (Kg/m^2^)	− 0.275	0.121	0.760	0.537	1.076
Follow-up (yr)	0.478	**0.002**	1.613	1.194	2.179
Osteoporosis	1.235	0.055	3.438	0.974	12.135
Pfirrmann	0.288	0.408	1.334	0.674	2.642
Distance*	− 1.942	< **0.001**	0.143	0.058	0.355
Sagittal screw angle*	2.695	< **0.001**	14.802	5.594	39.165
SS	− 0.021	0.723	0.979	0.872	1.100
PT	0.046	0.403	1.047	0.940	1.166

B, Regression coefficient; OR, Odds ratio; CI, Confidence interval; SS, Sacral slope; PT, Pelvic tilt; PI-LL, Pelvic incidence minus lumbar lordosis

Boldface values indicate *p* < 0.05

*Categorical variables

## Discussion

Although advances in surgical techniques have made spinal surgeries for patients safer and more extensive and complex, some scholars even think that it may be an effective way to prevent ASD to pull out the screw for patients with unsatisfactory pedicle screw implantation [16], identifying risk factors of pedicle screw internal fixation is still essential for patients [17, 18]. By observing imaging examination of outpatient follow-up patients after lumbar fusion, we found that compared with patients with back pain released significantly, patients with persistent low back pain showed a closer distance of lumbar pedicle screws apex to superior endplate with pedicle leaning cranially in a larger angle and seems to have higher incidence of proximal ASD, as was reported that degenerative change occurred in the proximal segment to the fusion levels [19–21]. In addition to known risk factors, such as age, BMI, adjacent disk degeneration, sagittal imbalance, length of fusion, years of follow-up and osteoporosis. To the authors’ knowledge, there were few studies analyzing the relationship between position of screw and incidence of ASD in lumbar spine. To explore the relationship and avoid the influence of fusion segment difference, we evaluated the incidence of ASD at L3–4 in patients performing PLF at L4-S1 with a least 2-years follow-up. After reviewing the collected data, we found the large cranial angled screw implantation and short distance of screw apex to superior endplate correlated with higher VAS, ODI score and higher incidence of ASD. As has been reported before, the pedicle screw orientation toward superior endplate increases the bending moments, jeopardizes screw’s fatigue life and puts the entire construct at the risk of failure [22, 23]. In long thoracic fusion to pelvis, upper instrumented vertebra pedicle screw-vertebra angle ≥ 3° was associated with 2.7-fold greater odds of proximal junctional kyphosis and 3.6-fold greater odds of proximal junctional failure compared with upper instrumented vertebra pedicle screw-vertebra angle < 3° [24]. Youssef demonstrated when pedicle screw angled toward superior vertebral endplate, a bending moment significantly increased, representing a concentration of axial load force on the tip of pedicle screw, and when the pedicle screw angled parallel to the upper endplate or angled caudally, it follows Saint–Venant’s principle, distributing the axial load over a greater surface area and reducing the bending moment on the screw [23]. Our study demonstrated stress on superior endplate is closely related to the position of the pedicle screws, although there is no encroachment of superior endplate. In model 1, sagittal screw angle and distance are highly reliable predictors of ASD, as the Hosmer–Lemeshow test and ROC curve of prognostic model indicate the model has effective discrimination and calibration. ROC curve of sagittal screw angle and distance of prediction of ASD showed patients with sagittal screw angle > 5.500° or distance < 6.250 mm were more likely to develop ASD. It is possible that stiffness is more easily transmitted to superior endplate when the pedicle screw apex is closer to the endplate, and the force acts more directly on the upper endplate along the direction of the screw under a bigger sagittal screw angle. Endplates are susceptible to trabecular microdamage in compression. So, it is possible that during flexion and extension of lumbar spine, the pressure on the vertebral endplate is more pronounced in patients with shorter distance and bigger sagittal angle of pedicle screw to the superior endplate. Over time, the increased stress on superior endplate is likely to lead to endplate deformation and collapse, affecting the transport of intervertebral disk nutrients, accelerating the degenerative changes of intervertebral disc, and leading to the occurrence of adjacent segment disease and low back pain. And the degenerative disk disease is often accompanied by changes of stress distribution in intervertebral disk [25]. Accordingly, though pedicle screws placed in a cranial-caudal trajectory may have greater pull-out strength than screws placed in a “straightforward” (parallel to the endplate) trajectory [26], a big cranial angled pedicle screw with close distance to superior endplate causes significantly greater stress in cortical bone than a smaller angled screw [27], which may enhance strength on adjacent disk and accelerate the degeneration of intervertebral disc, induce the development of ASD and cause worse clinical outcome, such as high low back VAS and ODI. In this study, we believe that controlling the distance between the screw and superior endplate and maintaining a small sagittal angle can effectively prevent the occurrence of ASD. However, for patients with severe osteoporosis, the anti-pull-out strength of pedicle screws should be considered, in the process of lumbar pedicle screw implantation, the angle between the screw and superior endplate can be appropriately increased to enhance the anti-pull-out strength, but the sagittal angle should not exceed 5.500° (L4 level), otherwise it will also increase the incidence of ASD.

There are also several limitations of this study. First, the muscles, skin, and other soft tissues are not included in the model. There are many other important factors that affect ASD, including age, BMI, length of fusion, type of instrumentation, sagittal parameters [28], and how these factors actually affect ASD needs to be clarified through future study. Second, as with any retrospective study, selection biases may be present. Finally, there is no consensus in assessing radiological or clinical ASD. Therefore, radiological and clinical results related to ASD can be influenced by the definition of ASD.

## Conclusion

Our results indicate that incidence of ASD increase with the prolongation of time after surgery, shorter distance of pedicle screw apex to the superior endplate and bigger sagittal screw angle between pedicle screw and superior endplate, especially with a distance < 6.250 mm or sagittal screw angle > 5.500°. Spinal surgeons should be aware of the placement of pedicle screws during surgery, and it may be an effective way to prevent ASD to pull out the screw for patients with unsatisfactory pedicle screw implantation.

## Data Availability

Data will be available upon request by the corresponding author on reasonable request.

## Abbreviations

ASD: Adjacent segment disease
PLF: Posterolateral lumbar fusion
BMI: Body mass index
VAS: Visual analogue scale
ODI: Oswestry disability index
Distance: The distance between distal screw and superior endplate
SS: Sacral slope
PT: Pelvic tilt
PI-LL: Pelvic incidence minus lumbar lordosis
